# Association of physical activity, smoking, and socioeconomic factors on health checkup participation in community-dwelling stroke survivors aged 50 years or older

**DOI:** 10.1186/s12889-023-15403-6

**Published:** 2023-03-15

**Authors:** Mina Park, Jung Soo Lee, Yeo Hyung Kim

**Affiliations:** Department of Rehabilitation Medicine, College of Medicine, The Catholic University of Korea, Seoul, Republic of Korea

**Keywords:** Stroke, Health checkup, Factors, Community-dwelling stroke survivor, EuroQol 5-Dimension questionnaire

## Abstract

**Background:**

We investigated the sociodemographic and health-related factors associated with health checkup participation in community-dwelling stroke survivors.

**Methods:**

Among participants of the Korea National Health and Nutrition Examination Survey, 642 stroke survivors were included. We investigated the sociodemographic, medical, and health-related quality of life factors—evaluated by the EuroQol 5-Dimension Questionnaire (EQ-5D)—associated with participation in any type of health checkup. To explore the associations between multiple variables and health checkup participation, a multivariable complex-sample logistic regression model was used.

**Results:**

One-third of the community-dwelling stroke survivors did not receive a health checkup in the past two years. Insufficient physical activity (OR: 0.5, 95% CI: 0.3–0.9), current smoking (OR: 0.4, 95% CI: 0.2–0.8), low education level (OR: 0.5, 95% CI: 0.3–0.9), living alone (OR: 0.5, 95% CI: 0.3–0.998), and no occupation (OR: 0.5, 95% CI: 0.3–0.9) showed independent negative associations with health checkup participation. Among the five EQ-5D dimensions, mobility, self-care, usual activities, and pain/discomfort dimensions were associated with health checkup participation rate.

**Conclusion:**

Policies and further research are needed to promote health checkups for stroke survivors who are physically inactive, currently smoking, living alone, unemployed, less educated, or having extreme problems in their daily lives.

## Background

Since the World Report on Disability was announced by the World Health Organization (WHO) in 2011, the importance of the right to health for persons with disabilities has risen worldwide [1]. The priority task in the health field for people living with disabilities is to strengthen preventive medical care to promote health with an emphasis on the role of health checkups. Health checkups refer to medical screening programmes such as counselling, physical examinations, laboratory tests, and radiologic examinations to improve an individual’s health through early detection of risk factors and diseases and enabling early treatment [2, 3]. In addition, the government can reduce overall healthcare costs through health checkup programmes [4]. However, the participation rate in health checkups of people with disabilities is still lower than that of the general population [5–7].

Stroke is one of the leading causes of death and acquired long-term disability in older people [8, 9]. Although the age-adjusted mortality owing to stroke has decreased over the past few decades, the prevalence of stroke is increasing annually as the older population increases [10]. Furthermore, the life expectancy of stroke survivors, even with minor ischaemic stroke, is still significantly lower than that of the general population [11]. Stroke survivors have physical, social, and mental impairments and disabilities as secondary conditions owing to the disease, which adversely affect overall health after stroke [12]. In Korea, the age-standardised prevalence of stroke is 1.37%, and one-third of non-hospitalised stroke survivors have disabilities [13, 14]. Therefore, it is essential to continuously check the health status of stroke survivors to manage the sequelae properly, prevent stroke recurrence, and enable stroke survivors to live with a minimal disability even though they have an impairment.

The factors known to be related to the lower health checkup participation rate of people living with disabilities are a younger age (30 to 39 years), a middle-low level of income, no existing spouse, no chronic diseases, very bad subjective health condition, and more dependent level of activities of daily living (ADL) [7]. Similarly, in the general older population, although regional differences exist, the major factors associated with higher health checkup compliance are a shorter period of education, no smoking, higher physical activity, better eating habits, better self-rated health, and better ADL [15]. However, there is currently no knowledge of the factors associated with health checkup engagement among community-dwelling stroke survivors.

To increase the health checkup engagement of stroke survivors, who account for a large portion of the people living with disabilities, it is indispensable to understand the factors affecting health checkup participation; however, studies on this issue are still lacking. In this study, we determined the sociodemographic and health-related factors associated with health checkup participation in community-dwelling middle-aged and older stroke survivors aged ≥ 50 years.

## Methods

### Study design and participants

The current cross-sectional study utilised the data from the Korea National Health and Nutrition Examination Survey (KNHANES) VI (2014–2015) and VII (2016–2018), operated by the Korea Centers for Disease Control and Prevention (KCDC). KNHANES applies a stratified, multistage, clustered probability sampling survey to collect nationally representative data on clinical information, health-related behaviours, and nutrition. KNHANES provides sampling weights computed by processing design weights, non-response adjustment, post-stratification, and trimming extreme weights. Detailed descriptions of the design and variables of KNHANES have been presented earlier [16, 17].

People who answered ‘yes’ to the question ‘Have you ever been diagnosed with stroke by a doctor’? were regarded as ‘stroke survivors’ [18]. Among the participants between 2014 and 2018 (n = 39,199), 642 stroke survivors (327 men and 315 women) who were aged ≥ 50 years and completed health interviews and examinations were included. The Institutional Review Board at the KCDC approved the protocol, and all participants signed informed consent forms. Since we analysed publicly accessible data, the Institutional Review Board of our hospital exempted this study from ethical approval.

### Variables

‘Health checkup participants’ were defined as those who responded ‘yes’ to the question ‘Have you had a checkup for your health in the past two years?’ The individuals who answered ‘no’ to the abovementioned question were categorised as ‘health checkup non-participants’. Any type of health checkup was included such as private comprehensive health checkups, workplace special health checkups, National Health Insurance general health checkups, and other free health checkups.

Education level (high school graduation or higher, middle school graduation or lower), household income quartiles (high, middle-high, middle-low, or low), type of health insurance (national health insurance, or Medical Aid), and smoking habits (current, past, or never). According to the answer to the question ‘How many people live together in your house?’, participants were classified as ‘living alone’ or ‘living together with cohabitants’. Participants were classified as employed or unemployed by the answer to the question, ‘Have you worked for more than one hour for income or worked as an unpaid family worker for more than 18 hours in the last week?’ Perceived health condition (good, normal, or bad) was recorded by the question, ‘How do you feel about your health at ordinary times?’ Excessive alcohol consumption was defined as > 20 g/day for men and > 10 g/day for women [19]. Individuals with disabilities were defined as those who responded ‘yes’ to the question ‘Are your daily or social activities limited owing to health problems or physical or mental disabilities’? [14].

Physical activity was evaluated by the Korean version of the WHO Global Physical Activity Questionnaire, which assesses aerobic activities by collecting the time spent during a typical week [20, 21]. WHO Global recommendations on physical activity for health defines sufficient weekly physical activity level as follows: ≥ 150 min of moderate-intensity activity or ≥ 75 min of vigorous-intensity activity or an equivalent combination of moderate- and vigorous-intensity activity [18, 22]. Participants’ health-related quality of life was evaluated by the Korean version of the EuroQol 5-Dimension Questionnaire (EQ-5D-3 L) [23, 24]. The EQ-5D is a measure to assess health status in five dimensions (mobility, self-care, usual activities, pain/discomfort, and anxiety/depression) with three severity levels (no, some, or extreme problems) in each dimension.

The number of chronic diseases related to vascular risk factors for stroke including hypertension, diabetes, and hypercholesterolemia was calculated [25]. Individuals with systolic blood pressure ≥ 140 mmHg or diastolic blood pressure ≥ 90 mmHg or those taking antihypertensive drugs were regarded as having hypertension. The people with fasting blood glucose ≥ 126 mg/dL or HbA1c ≥ 6.5%, or those diagnosed with diabetes by a doctor or treated with hypoglycaemic drugs or insulin were considered to have diabetes. The presence of hypercholesterolemia was determined by total cholesterol ≥ 240 mg/dL or by taking cholesterol-lowering drugs. Obesity was defined as body mass index ≥ 25.0 kg/m^2^ in accordance with the WHO’s guidelines for the Asia-Pacific region [26].

### Statistical analysis

Participants’ characteristics were analysed by complex-sample descriptive statistics. The association of sociodemographic variables, health-related factors, and EQ-5D levels with health checkup participation was evaluated by complex-sample chi-square tests. The categorical data are presented as weighted percentages (standard errors of percentages). Multivariable complex-sample logistic regression analyses were used to explore the association between multiple variables and health checkup participation. The results of logistic regression analyses are presented as odds ratios (ORs) and 95% confidence intervals (CIs). To reflect the entire South Korean population, sampling weights provided by KNHANES were applied in all statistical analyses. Complex-sample procedures of SPSS version 24 (IBM/SPSS Inc., NY, USA) were used. Ps < 0.05 were deemed significant.

## Results

The health-related factors and sociodemographic characteristics of 642 stroke survivors are shown in Table 1. The weighted mean age was 68.1 years (SE = 0.4%). Nearly two-thirds (63.3%, SE = 2.1%) of the community-dwelling stroke survivors received a health checkup in the past two years. One-third of stroke survivors participated in sufficient physical activity. Two-thirds of stroke survivors reported no disabilities. However, half of the stroke survivors reported their perceived health as bad. Two-fifths were obese (40.8%, SE = 2.3%).

**Table 1:** Participant characteristics by health checkup participation status

Variables, % (SE^a^)	Total participants (N^b^ = 642)	Health checkup non-participants (n^b^ = 237)	Health checkup participants (n^b^ = 405)	*P* ^c^
**Age (years)**				0.506
50–59	20.9 (2.1)	20.8 (3.6)	21.0 (2.6)	
60–69	30.3 (2.2)	28.8 (3.6)	31.2 (2.7)	
70–79	36.4 (2.2)	35.0 (3.7)	37.3 (2.8)	
≥ 80	12.4 (1.5)	15.5 (2.6)	10.6 (1.8)	
**Sex**				0.310
Women	46.6 (2.3)	49.8 (3.9)	44.7 (2.8)	
Men	53.4 (2.3)	50.2 (3.9)	55.3 (2.8)	
**Education**				0.009
High school graduation or higher	27.6 (2.0)	20.3 (3.1)	31.7 (2.7)	
Middle school graduation or lower	72.4 (2.0)	79.7 (3.1)	68.3 (2.7)	
**Income**				0.524
High	11.7 (1.6)	9.6 (2.3)	12.9 (2.2)	
Middle-high	18.4 (2.0)	17.4 (3.2)	18.9 (2.5)	
Middle-low	25.7 (2.1)	24.4 (3.5)	26.5 (2.6)	
Low	44.2 (2.3)	48.6 (3.9)	41.6 (2.9)	
**Health insurance**				0.049
National health insurance	90.3 (1.4)	87.1 (2.4)	92.2 (1.6)	
Medical Aid	9.7 (1.4)	12.9 (2.4)	7.8 (1.6)	
**Living alone**				< 0.001
No	80.1 (1.7)	71.1 (3.4)	85.4 (1.8)	
Yes	19.9 (1.7)	28.9 (3.4)	14.6 (1.8)	
**Occupation**				0.001
Employed	30.3 (2.2)	20.8 (3.4)	35.7 (2.7)	
Unemployed	69.7 (2.2)	79.2 (3.4)	64.3 (2.7)	
**Obesity**				0.375
Yes	40.8 (2.3)	38.0 (3.7)	42.3 (3.0)	
No	59.2 (2.3)	62.0 (3.7)	57.7 (3.0)	
**Alcohol drinking**				0.789
Non-excessive	91.9 (1.4)	92.4 (2.1)	91.7 (1.8)	
Excessive	8.1 (1.4)	7.6 (2.1)	8.3 (1.8)	
**Smoking**				0.030
Never	49.6 (2.2)	51.0 (4.0)	48.8 (2.9)	
Past	33.8 (2.1)	27.1 (3.6)	37.6 (2.7)	
Current	16.6 (1.8)	21.9 (3.4)	13.6 (2.0)	
**Physical activity**				0.001
Sufficient	32.8 (2.2)	22.4 (3.3)	38.9 (3.0)	
Insufficient	67.2 (2.2)	77.6 (3.3)	61.1 (3.0)	
**Self-reported disabilities**				0.007
No	67.8 (2.4)	60.5 (3.7)	72.0 (2.7)	
Yes	32.2 (2.4)	39.5 (3.7)	28.0 (2.7)	
**Number of chronic diseases**				0.511
0	15.9 (1.9)	12.4 (3.0)	17.9 (2.4)	
1	37.0 (2.4)	40.0 (3.9)	35.2 (2.9)	
2	32.5 (2.3)	32.6 (3.9)	32.5 (3.0)	
3	14.6 (1.8)	14.9 (3.1)	14.4 (2.2)	
**Perceived health**				0.012
Good	9.8 (1.5)	7.1 (2.1)	11.4 (1.9)	
Normal	38.7 (2.2)	32.6 (3.3)	42.3 (2.8)	
Bad	51.4 (2.3)	60.2 (3.6)	46.4 (2.8)	

^a^SE, standard error

^b^Unweighted number

^c^*P*-values by complex-sample chi-square test comparing health checkup non-participants and health checkup participants

### Factors associated with health checkup participation

The associations of sociodemographic and health-related factors and health checkup engagement are shown in Table 1. Among sociodemographic factors, health checkup non-participants had significantly higher percentages of lower education (79.7% vs. 68.3%; *P =* 0.009), Medical Aid (12.9% vs. 7.8%; *P =* 0.049), living alone (28.9% vs. 14.6, *P* < 0.001), and unemployed (79.2% vs. 64.3%, *P =* 0.001) than health checkup participants. Among health-related factors, health checkup non-participants showed significantly higher percentages of current smoking (21.9% vs. 13.6%, *P =* 0.030), insufficient physical activity (77.6% vs. 61.1%, *P =* 0.001), disabilities (39.5% vs. 28.0, *P =* 0.007), and bad perceived health (60.2% vs. 46.4%, *P =* 0.012) than health checkup participants. In addition, there was no association of health checkup rate with age, sex, income, obesity, alcohol drinking, or the number of chronic diseases.

Table 2 shows the unadjusted and adjusted correlates of health checkup participation among community-dwelling stroke survivors. Lower education level (OR: 0.5, 95% CI: 0.3–0.9), Medical Aid (OR: 0.6, 95% CI: 0.3–0.996), living alone (OR: 0.4, 95% CI: 0.3–0.6), unemployed status (OR: 0.5, 95% CI: 0.3–0.7), current smoking (OR: 0.4, 95% CI: 0.2–0.8), insufficient physical activity (OR: 0.5, 95% CI: 0.3–0.7), and disabilities (OR: 0.6, 95% CI: 0.4–0.9) were significantly associated with health checkup participation. The significant associations of education level, living alone, occupational status, smoking habits, physical activity, and health checkup participation remained significant after adjusting for multiple confounders. Stroke survivors with lower education were less likely to receive health checkups than those with higher education (adjusted OR: 0.5, 95% CI: 0.3–0.9). Participants living alone (adjusted OR: 0.5, 95% CI: 0.3–0.998) or unemployed (adjusted OR: 0.5, 95% CI: 0.3–0.9) showed independent lower compliance to health checkups than those living with cohabitants or employed. Stroke survivors who did not participate in sufficient physical activity showed a tendency not to receive health checkups (adjusted OR: 0.5, 95% CI: 0.3–0.9). Participants who were currently smoking had significantly fewer health checkups than those who had quit smoking (adjusted OR: 0.4, 95% CI: 0.2–0.8).

**Table 2:** Unadjusted and adjusted odds ratios for health checkup participation in community-dwelling stroke survivors

Variables, OR^a^ (95% CI^b^)	Unadjusted OR (95% CI)	Adjusted OR^c^ (95% CI)
**Age (years)**		
50–59	Reference	Reference
60–69	1.1 (0.6–1.9)	1.1 (0.5–2.4)
70–79	1.1 (0.6–1.9)	1.5 (0.7–3.2)
≥ 80	0.7 (0.3–1.3)	1.0 (0.4–2.8)
**Sex**		
Women	Reference	Reference
Men	1.2 (0.8–1.8)	0.9 (0.5–1.8)
**Education**		
High school graduation or higher	Reference	Reference
Middle school graduation or lower	0.5 (0.3–0.9)	0.5 (0.3–0.9)
**Income**		
High	Reference	Reference
Middle-high	0.8 (0.4–1.8)	1.3 (0.5–3.3)
Middle-low	0.8 (0.4–1.7)	1.7 (0.7–4.2)
Low	0.6 (0.3–1.3)	2.0 (0.8–5.1)
**Health insurance**		
National health insurance	Reference	Reference
Medical Aid	0.6 (0.3–0.996)	0.8 (0.4–1.8)
**Living alone**		
No	Reference	Reference
Yes	0.4 (0.3–0.6)	0.5 (0.3–0.998)
**Occupation**		
Employed	Reference	Reference
Unemployed	0.5 (0.3–0.7)	0.5 (0.3–0.9)
**Obesity**		
No	Reference	Reference
Yes	1.2 (0.8–1.8)	1.6 (1.0–2.5)
**Alcohol drinking**		
Non-excessive	Reference	Reference
Excessive	1.1 (0.5–2.4)	1.1 (0.5–2.7)
**Smoking**		
Never	0.7 (0.4–1.1)	0.6 (0.3–1.3)
Past	Reference	Reference
Current	0.4 (0.2–0.8)	0.4 (0.2–0.8)
**Physical activity**		
Sufficient	Reference	Reference
Insufficient	0.5 (0.3–0.7)	0.5 (0.3–0.9)
**Disabilities**		
No	Reference	Reference
Yes	0.6 (0.4–0.9)	0.8 (0.5–1.3)
**Number of chronic diseases**		
0	Reference	Reference
1	0.6 (0.3–1.2)	0.6 (0.3–1.1)
2	0.7 (0.3–1.4)	0.6 (0.3–1.4)
3	0.7 (0.3–1.5)	0.6 (0.2–1.5)
**Perceived health**		
Good	Reference	Reference
Normal	0.8 (0.4–1.7)	0.9 (0.4–2.3)
Bad	0.5 (0.2–1.002)	0.7 (0.3–1.6)

^a^OR, odds ratio

^b^CI, confidence intervals

^c^Adjusted for all other variables in column

### Association between health-related quality of life and health checkup participation

The proportion of stroke survivors who reported no problems in each dimension of the EQ-5D (mobility, self-care, usual activities, pain/discomfort, and anxiety/depression) were 51.2% (SE = 2.3%), 74.2% (SE = 2.1%), 61.5% (SE = 2.3%), 51.4% (SE = 2.5%), and 75.5% (SE = 2.1%), respectively. Extreme problems in the five dimensions were reported by 3.7% (SE = 0.8%), 3.7% (SE = 0.8%), 6.0% (SE = 1.2%), 9.8% (SE = 1.5%), and 3.4% (SE = 0.9%) of stroke survivors, respectively.

**Fig. 1:**
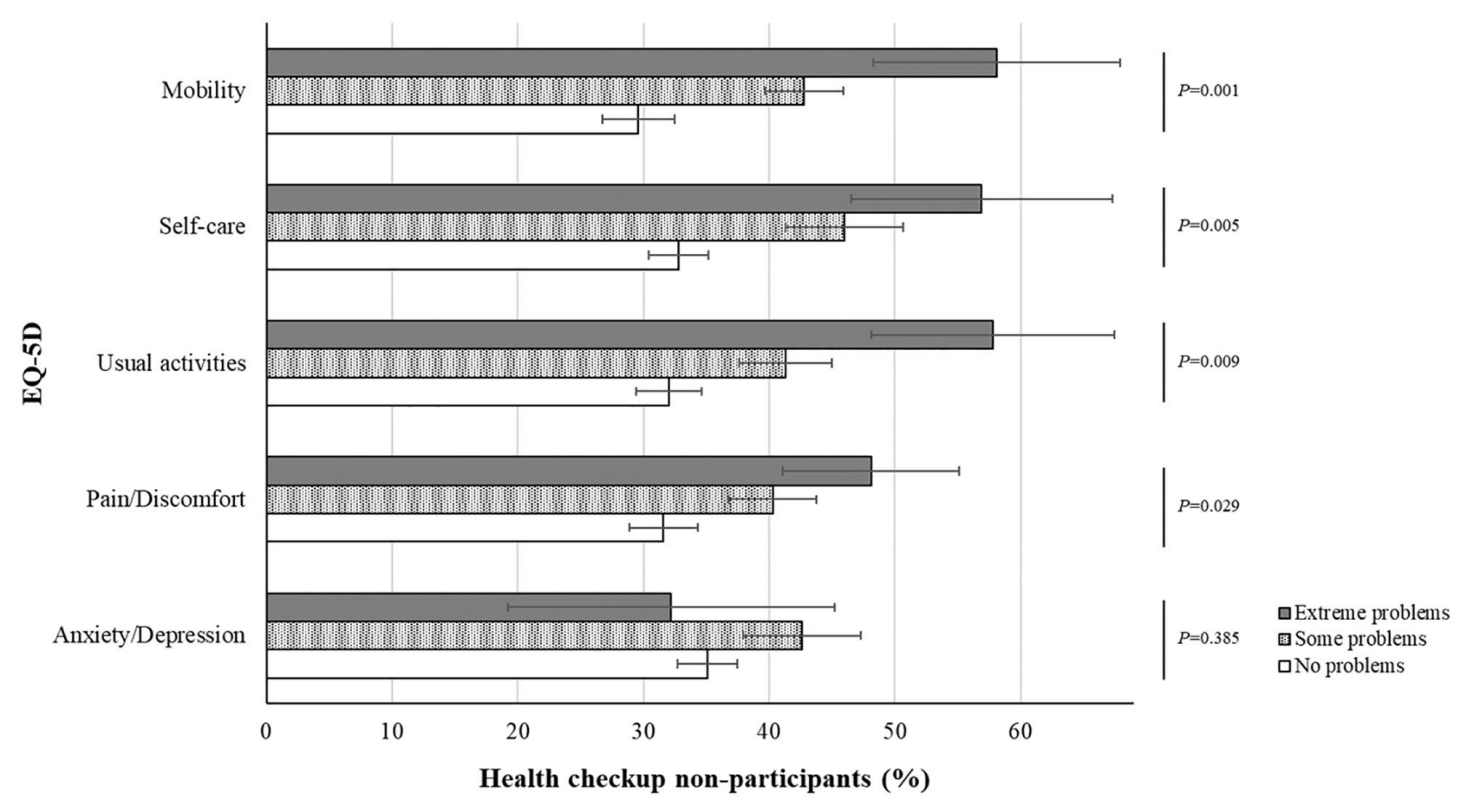
Proportion of health checkup non-participants in each EQ-5D-3 L dimension. EQ-5D, EuroQol 5-Dimension Questionnaire

Figure 1 shows the associations between the five EQ-5D dimensions and health checkup participation rate. Participants with problems in mobility, self-care, usual activities, and pain/discomfort dimensions received less health checkups than their counterparts. More severe problems in mobility, self-care, usual activities, and pain/discomfort dimensions were associated with lower health checkup rates. In contrast, the severity of the anxiety/depression dimension was not associated with the health checkup rate.

## Discussion

The present nationwide study demonstrated that the major factors related to health checkup participation in community-dwelling stroke survivors were education level, living alone, occupational status, smoking habits, and degree of physical activity. Stroke survivors with problems in mobility, self-care, usual activity, or pain/discomfort tended to engage in less health checkups. Therefore, greater attention should be paid to community-dwelling stroke survivors with low education, living alone, unemployed, currently smoking, insufficient physical activity, or problems in self-care and usual activities to lead them to participate in health checkups.

The stroke survivors with insufficient physical activity had a 0.5-fold lower odds of health checkup engagement than those with sufficient physical activity after adjusting for multiple potential confounders. These results are consistent with an earlier study that found that performing daily exercise was positively related to health checkups in the general population in Japan [27]. However, the association in stroke survivors has not been found in other studies. A sedentary lifestyle and low physical activity have been shown to increase the risk of incident and recurrent stroke [28]. Therefore, physical activity level—as an independent factor associated with health checkups in community-dwelling stroke survivors and as an established risk factor for cardiovascular disease—may be a key factor to determine people in need of regular health checkups.

Current smoking status showed an independent negative association with health checkup participation in stroke survivors. The similar association between smoking and non-participation in health checkups has been reported in older populations in a previous study [29]. An interesting result related to smoking was that past smokers showed a higher health checkup rate than never smokers as well as current smokers. A possible explanation is that people who successfully quit smoking are aware of the importance of their health and have the will to actively prevent diseases. After quitting, the elevated risk of stroke owing to smoking declines and disappears after five years [30]. Therefore, providing smoking-related counselling to stroke survivors may be a key public health intervention to promote health checkups and prevent recurrent stroke.

Among the sociodemographic factors, having an occupation and living with cohabitants were independent factors and positively associated with stroke survivors’ health checkup rate. Previous studies of older adults and the general population have found consistent results—that occupation was related to health checkup behaviour [31, 32]. In Korea, for the health promotion of workers, a regular health checkup is mandatory for office workers once every two years and for non-office workers once a year. This legal obligation might have an impact on the higher health checkup participation rate in stroke survivors with a job. However, because KNHANES did not collect information about individuals’ retirement, we could not analyse differences between retired and long-term unemployed participants, despite that many participants were within retirement age.

The results of our study showed that low education level was associated with low health checkup compliance, which is inconsistent with an earlier study conducted in Japan that reported an association between a shorter educational period and higher health checkup participation [15]. This inconsistency may stem from the demographic, medical, or social differences among the study participants, who were recruited from different regions or countries across the studies. The support of people with high social contact, such as the presence of cohabitants, seems to be a crucial factor leading to participation in health examinations. In a previous study that included participants with all types of disabilities, family factors such as having a spouse had a significant effect on the health checkup rate [7]. Sociodemographic factors such as occupation, social contact, and education are difficult to change by an individual’s effort. However, the importance of clarifying these unmodifiable factors stems from the possibility of providing focused help. The government or healthcare professionals should increase the emphasise the importance of health checkups among non-hospitalised stroke survivors without an occupation, living alone, or with lower education.

We additionally analysed the association between health-related quality of life and health checkup participation. The non-participation rate was higher in the groups reporting extreme problems in dimensions related to ADL such as mobility, self-care, and usual activities. These results are consistent with earlier studies that the people with dependent ADL had a low health checkup rate as compared to their counterparts [7, 33]. Stroke survivors with difficulties in mobility, self-care, and usual activities will have difficulty living independently and need help from others in daily life. Individuals in such conditions can participate in health checkups only if they have extra personnel who can help them move to the hospital and require extra effort and time to examine in the hospital. We suggest that to increase the health checkup participation of people with severe problems in ADL, it is necessary to provide special care, such as a home visit programme, health checkup institutions dedicated only to the people with severe disabilities, or programmes/expanding programmes that provide transportation for individuals who require mobility assistance.

This was the first study to investigate the independent factors associated with health checkup participation in community-dwelling stroke survivors. We examined multiple demographic, socioeconomic, and medical variables, along with health-related quality of life. Furthermore, the results were based on nationally representative data. Nevertheless, there are several limitations. First, a causal relationship between the multiple factors and observed trends cannot be inferred from this cross-sectional study. Second, sample selection bias and endogenous bias may exist. Since our study only includes data from Korea, an endogenous bias regarding ethnic homogeneity and sociality may exist. International and multi-ethnic studies are needed for more comprehensive results. Third, since the KNHANES survey did not target only stroke survivors, information on stroke subtype or severity, which can be an additional confounding factor, is lacking.

## Conclusion

Insufficient physical activity, current smoking, low education level, living alone, and without occupation were the independent factors associated with poor health checkup participation among community-dwelling stroke survivors aged 50 years or older. Social protection to induce health checkups are needed for health equity of stroke survivors in those conditions. Furthermore, health checkup programmes should be expanded according to the severity of impairment and disability-friendly health checkup institutions should be considered to strengthen stroke survivors’ access to medical services.

## Data Availability

The KNHANES database is publicly available (https://knhanes.kdca.go.kr [in Korean]).

## Abbreviations

ADL: Activities of daily living
EQ-5D: EuroQol 5-Dimension Questionnaire
KCDC: Korea Centers for Disease Control and Prevention
KNHANES: Korea National Health and Nutrition Examination Survey
WHO: World Health Organization

## References

[1] World Health Organization. Summary: world report on disability 2011. 2011. 26131540

[2] Hozawa A, Kuriyama S, Watanabe I, Kakizaki M, Ohmori-Matsuda K, Sone T, Nagai M, Sugawara Y, Nitta A, Li Q (2010). Participation in health check-ups and mortality using propensity score matched cohort analyses. Prevent Med.

[3] Kang HT (2022). Current status of the national health screening programs in South Korea. Korean J Fam Med.

[4] Shin DW, Cho J, Park JH, Cho B (2022). National general health screening program in Korea: history, current status, and future direction. Precis Future Med.

[5] Tezzoni LI, McCarthy EP, Davis RB, Harris-David L, O’Day B (2001). Use of screening and preventive services among women with disabilities. Am J Med Qual.

[6] Park J-H, Lee J-S, Lee J-Y, Gwack J, Park J-H, Kim Y-I, Kim Y (2009). Disparities between persons with and without disabilities in their participation rates in mass screening. Eur J Public Health.

[7] Chun SM, Hwang B, Park J-H, Shin H-I (2012). Implications of sociodemographic factors and health examination rate for people with disabilities. Arch Phys Med Rehab.

[8] Hackett ML, Duncan JR, Anderson CS, Broad JB, Bonita R (2000). Health-related quality of life among long-term survivors of stroke: results from the Auckland Stroke Study, 1991–1992. Stroke.

[9] Mozaffarian D, Benjamin EJ, Go AS, Arnett DK, Blaha MJ, Cushman M, Das SR, De Ferranti S, Després J-P, Fullerton HJ. Heart disease and stroke statistics—2016 update: a report from the American Heart Association. Circulation. 2016;133(4):e38–360. 10.1161/CIR.0000000000000350 26673558

[10] Hong KS, Bang OY, Kang DW, Yu KH, Bae HJ, Lee JS, Heo JH, Kwon SU, Oh CW, Lee BC (2013). Stroke statistics in Korea: part I. Epidemiology and risk factors: a report from the korean stroke society and clinical research center for stroke. J Stroke.

[11] Prencipe M, Culasso F, Rasura M, Anzini A, Beccia M, Cao M, Giubilei F, Fieschi C (1998). Long-term prognosis after a minor stroke: 10-year mortality and major stroke recurrence rates in a hospital-based cohort. Stroke.

[12] Kim P, Warren S, Madill H, Hadley M (1999). Quality of life of stroke survivors. Qual Life Res.

[13] Kim JY, Kang K, Kang J, Koo J, Kim DH, Kim BJ, Kim WJ, Kim EG, Kim JG, Kim JM (2019). Executive summary of stroke statistics in Korea 2018: a report from the Epidemiology Research Council of the korean Stroke Society. J Stroke.

[14] Ju YW, Lee JS, Choi Y-A, Kim YH. Causes and trends of disabilities in community-dwelling stroke survivors: a population-based study.Brain Neurorehabil. 2022;15(1). 10.12786/bn.2022.15.e5 PMC9833459 36743839

[15] Mitsuhashi Y, Kishi R, Eguchi T, Miyake H, Maeda N (2003). Factors associated with participation in medical checkups of the elderly at home comparison of 3 regions with different social backgrounds. [Nihon Koshu Eisei Zasshi]. Jpn J Public Health.

[16] Kweon S, Kim Y, Jang MJ, Kim Y, Kim K, Choi S, Chun C, Khang YH, Oh K (2014). Data resource profile: the Korea National Health and Nutrition Examination Survey (KNHANES). Int J Epidemiol.

[17] Lee JS, Kim YH (2020). Vitamin D status and related factors among korean stroke survivors: a nationwide population-based study. J Nutr Sci Vitaminol.

[18] Choi YA, Lee JS, Park JH, Kim YH (2022). Patterns of physical activity and sedentary behavior and their associated factors among nondisabled stroke survivors. Maturitas.

[19] Park JT, Kim BG, Jhun HJ (2008). Alcohol consumption and the CAGE questionnaire in korean adults: results from the Second Korea National Health and Nutrition Examination Survey. J Korean Med Sci.

[20] Armstrong T, Bull F (2006). Development of the World Health Organization Global Physical Activity Questionnaire (GPAQ). J Public Health.

[21] Lee J, Lee C, Min J, Kang DW, Kim JY, Yang HI, Park J, Lee MK, Lee MY, Park I (2020). Development of the Korean Global Physical Activity Questionnaire: reliability and validity study. Glob Health Promot.

[22] World Health Organization (2010). Global recommendations on physical activity for health.

[23] Kim SH, Hwang JS, Kim TW, Hong YS, Jo M-W (2012). Validity and reliability of the EQ-5D for cancer patients in Korea. Support Care Cancer.

[24] Kwon KM, Lee JS, Jeon NE, Kim YH (2017). Factors associated with health-related quality of life in Koreans aged over 50 years: the fourth and fifth Korea National Health and Nutrition Examination Survey. Health Qual Life Outcomes.

[25] Kleindorfer DO, Towfighi A, Chaturvedi S, Cockroft KM, Gutierrez J, Lombardi-Hill D, Kamel H, Kernan WN, Kittner SJ, Leira EC (2021). 2021 guideline for the prevention of stroke in patients with stroke and transient ischemic attack: a guideline from the American Heart Association/American Stroke Association. Stroke.

[26] World Health Organization. The Asia-Pacific perspective: redefining obesity and its treatment. 2000.

[27] Otsuka T, Konta T, Sho R, Osaki T, Souri M, Suzuki N, Kayama T, Ueno Y (2021). Factors associated with health intentions and behaviour among health checkup participants in Japan. Sci Rep.

[28] Eijsvogels TM, Molossi S, Lee D-C, Emery MS, Thompson PD (2016). Exercise at the extremes: the amount of exercise to reduce cardiovascular events. J Am Coll Cardiol.

[29] Yamaguchi M, Yoshida T, Yamada Y, Watanabe Y, Nanri H, Yokoyama K, Date H, Miyake M, Itoi A, Yamagata E (2018). Sociodemographic and physical predictors of non-participation in community based physical checkup among older neighbors: a case-control study from the Kyoto-Kameoka longitudinal study, Japan. BMC Public Health.

[30] Wolf PA, D’Agostino RB, Kannel WB, Bonita R, Belanger AJ (1988). Cigarette smoking as a risk factor for stroke: the Framingham Study. JAMA.

[31] Noguchi R, Shen J (2019). Factors affecting participation in health checkups: evidence from japanese survey data. Health Pol.

[32] Okura M, Ogita M, Yamamoto M, Nakai T, Numata T, Arai H (2018). Health checkup behavior and individual health beliefs in older adults. Geriatr Gerontol Int.

[33] Chan L, Doctor JN, MacLehose RF, Lawson H, Rosenblatt RA, Baldwin L-M, Jha A (1999). Do Medicare patients with disabilities receive preventive services? A population-based study. Arch Phys Med Rehab.

